# Foreign Summary

**Published:** 1839-05-11

**Authors:** 


### FOREIGN SUMMARY.

*A Lecture on Inflammation.* By Professor Magendie.—You may have remarked that I avoid sedulously the use of the word *inflammation*, except when about to point out the erroneous notions it involves. Yet it expresses something. If its sole defect were to have been ill-chosen, to have been based on incorrect metaphor, the repugnance I feel for it would be puerile, and more worthy of the pedant than the physiologist,—but such is not the case. You know as well as I do, how erroneous are the theories represented by the word, and how various authors in different countries have laboured to add new fallacies to the already abundant stock. Before recurring to these, let me briefly enumerate the principal phenomena that occur in the substance of tissues said to be inflamed. You can easily ascertain them for yourselves with the microscope.

In order to appreciate correctly the modifications that occur in the capillary circulation, it is necessary that we should first understand thoroughly its normal condition; otherwise we should commit no few errors, just as others have done before us. This small animal is tightly fixed to a plate of cork; and the eye, aided by a microscope, follows the course of the globules through the infinitely minute capillary tubes of the mesentery. Apply an alkali or any other chemical agent to any point of the membrane, and immediately the circulation is observed to stop there; nothing is thenceforth perceptible in that situation but an obscure and motionless spot, and around this the capillary vessels are swollen. It is evident that the blood which should have passed through the obliterated vessels, regurgitates into the neighbouring tubes. What I have now said of chemical agents is equally applicable to every substance which modifies the texture of the vessels, or the degree of compression of the liquids. From the moment a want of harmony between the diameter of the capillaries and the volume of the molecules of the blood is brought about obstruction supervenes, and then follow the appearances ascribed to inflammation. Let us next see into the consequences of the stoppage of the circulation.

Inasmuch as a greater quantity of blood passes in a given time through such vessels as remain free in their interior, those vessels contain a great number of globules, and their colour consequently becomes deeper. This explains to you why inflamed tissues redden; it may also happen that blood finds its way into vessels ordinarily traversed by white fluids only; of this you have a familiar example in conjunctivitis.

The increase of pressure exercised on the inner wall of the capillaries, necessarily causes their dilatation. Their coats swell, fill a larger space than before, and give passage through their widened pores, either to the blood in substance, or to some of its materials. These phenomena of dilatation and extravasation produce more or less tumefaction of the part where they occur. The swelling reaches its maximum in the centre, for there all motion on the part of the liquids is suspended. At the circumference, where the blood continues to move, but in columns of larger diameter than usual, the swelling is less marked, and diminishes in proportion as we make our examination further from the inflamed point. Accordingly, you will remark that inflammatory swellings are generally conical, the most prominent point corresponding to the centre of the tumour, for there the obstruction is complete.

As the temperature of the body depends on the passage of the blood through the tissues, it is evident that it will increase in the direct ratio of the volume of the liquid. We have just seen that inflammation is accompanied by the accumulation of a greater quantity of blood than usual in the vessels, the animal heat of an inflamed part is, therefore, notably increased; this fact cannot escape the observation of any one who examines a part in that condition. The patient himself is conscious of the elevation of temperature. At the same time that the inflamed point is red, swelled, and hot, it becomes the seat of acute or dull pain, according to the nature of the tissue affected. Indeed, in the case of the ligaments, tendons, and cartilages, pain may be completely wanting, and its character varies exceedingly under different circumstances. The mode of distribution of the nervous filaments, their abundance or rarity, the presence or absence of an investing aponeurosis, explain the infinite varieties of sensibility observed in inflamed tissues. The nerves which ramify between the interstices of the different organs, and expand in the substance of their parenchymata, are compressed by the swelling of the vessels, and by the matters effused from their interior; it is they that transmit to the brain the impression of pain. It is possible, too, that the vascular coats themselves, according as they are, or are not, traversed by blood, become painful.

Inflamed parts frequently present unusual pulsations, whence it has been inferred that the vitality itself of the vessels is modified: the patient is conscious of these pulsations. But, I would ask, have the mechanical modifications undergone in this case by the local circulation been allowed their due importance? Whenever an artery is closed at one end, and so presents an insurmountable resistance to the passage of the fluids into the capillary rete, its coats support very considerable pressure, and dilate under each contraction of the heart. Now, this is the state of things in an inflamed part. The nerves, compressed at the instant each arterial pulsation takes place, transmit the impression to the brain; the phenomenon now alluded to is, therefore, physical in essence, though vital in its results,—the first cause of all the disorders observed is the obstruction of the capillary vessels.

In this way the analysis of the course of the blood in an inflamed part explains the true signification of the famous words—*dolor, calor, tumour, rubor.* The pain results from the compression of the nervous filaments by the obliterated or distended vessels; the heat shows that the blood passes in greater abundance into the neighbouring capillaries which continue to be permeable; the swelling is occasioned by the dilatation of the vessels, and by the extravasation of the materials of the blood; the redness depends on the presence of an unusual quantity of globules.

Now, in all this there is not a word about *irritation*, or *organic contractility*, or *insensible sensibility become sensible*, or *spontaneous movements of the globules*; and, in truth, such explanations of the phenomena are utterly deceptive. There is one experiment, in particular, which is cited as conclusive, and has for centuries served as the basis of numerous medical doctrines. We are told that when the mesentery of a living animal is pricked with a needle, the blood flows to the injured point from all sides. Why should the currents of blood change their direction and become retrograde in some of the capillaries of the part? There must be some force that attracts them, and this a stronger force than that of the heart itself, inasmuch as the globules often move in the opposite direction to that in which the action of that organ tends to propel them. This newly-developed force is *irritation*. You have, by your experiment, *stimulated* a point of the membrane, perverted its vitality, and so drawn thereto the blood contained in the surrounding vessels; all these phenomena are the result of irritation. Ere long you see the tissues swell, redden, grow hot, and painful, and extravasation occur in the vicinity,—all this is the result of a new process called *inflammation*. Call irritation *stimulus*, and inflammation *fluxus*, and you restore to its ancient importance the memorable axiom, *ubi stimulus, ibi fluxus*. Yet certain persons, forgetting these ideas, which are as old as medicine itself, talk to us of a new medical doctrine based on *physiology*.

Gentlemen, I have performed this experiment as well as others, and you shall learn the result. When one chances to prick a capillary vessel of the mesentery, the blood escapes by the opening, and the globules of the neighbouring tubes rush to the orifice produced, no matter what was the direction of their previous course. If, on the contrary, the point of your instrument simply enters the tissue of the membrane, without injuring any of its capillaries, the circulation continues as before: no abnormal movements of the globules are produced. These results merit serious examination. What, inflammation depends, you allege, on the exaltation of the vitality of a part, and here I find that pricking an artery determines an afflux of blood, while similarly injuring a membrane produces no derangement in the course of the fluid! And this, although the sensibility of the artery is positively null, in comparison with that enjoyed by the membrane in which it ramifies. If your theory were well founded, the violence of the inflammatory phenomena should be in harmony with the degree of vitality of the tissues: experience shows the precise contrary to be the truth. *Irritation* (to speak your language) is ineffectual in really *irritable* parts; powerful in those deprived of *irritability*. But, such absurd and contradictory hypotheses as these are, I delight to perceive, beginning to sink into merited contempt. In a variety of essays recently published, among others, that of Todd, of Liverpool, the question of inflammation is quite otherwise considered than has heretofore been the habit, and accurate microscopical researches substituted for rude and superficial observations.

But to the true explanation of the phenomena I have described:—When you pierce the wall of a capillary vessel, the blood, meeting with less resistance at the divided point than elsewhere, escapes through it, and flows out as long as the elasticity of the vascular tunics is not exhausted. But we know that the wounded capillary does not ramify by itself, but communicates by a multitude of branches with the neighbouring vessels of the same description, and that the pressure is equally divided among all. When you act on one of these vessels, you act at the same time on all the rest. The most distant globules tend as strongly to escape by the orifice you have made as those in its immediate vicinity, because an equilibrium tends to become established. But the blood, we are told, has been seen to go backwards: to direct itself from the veins towards the arteries. Not a doubt of it; it would be marvelous were it otherwise; for, from the moment the resistance diminishes in any point, the liquid must, by virtue of the laws of physics, flow thereto from every possible quarter. Distend an India-rubber tube with water, and then make a hole in its centre, and you will find that the contained fluid reaches the opening from both ends with equal facility. The elasticity of its walls explains the phenomenon fully; and the same mechanism is in play in the living vessels. The globules are sometimes observed to suddenly reassume their natural course; when this is the case, by examining the point of the vessel injured, you will find that a clot has formed, and obliterated the opening; remove this clot, and the disordered movements instantly recommence.

The course of the blood is not modified when the membrane only is punctured, simply because the properties of the blood are unchanged thereby, and no solution of continuity effected in its tubes,—the impelling agent remains the same as before; there is no reason why any modification of the circulation should ensue.

If, instead of opening the vessel, you apply an acid to its outer surface, phenomena of a different nature follow; but these depend wholly on chemical action, as has been proved by M. Leuret in a series of most interesting essays. The acid combines with the tissue, renders it tough and hard, by taking up its water, and so diminishes the diameter of the capillary vessel, which becomes too narrow proportionally to the volume of the globules; these stagnate and become motionless behind the contracted point. The blood which should have passed through the spot in question, flows back into the surrounding vessels, dilates them, and makes them appear larger than they previously were. In this case, as in the former, these artificial irritations and inflammations are nothing more than mechanical results. And yet these are the experiments which serve as the basis of the theory of irritation and inflammation,—a theory in which nothing is taken into consideration but the modified vitality of the tissues. I am well aware that at the present day it has lost something of its pristine vogue, but it still enjoys a certain share of credit with some practitioners, and it is well to annihilate even its last vestiges of popularity.

I have by no means exhausted the list of superannuated hypotheses, wherewith attempts have been made to explain the nature of inflammatory pneumonia. Take, for instance, the notions propounded by Döllinger, in Germany: this author commences a recent essay by establishing that the globules are so many little beings, independent of the action of the heart, moving where they will, and having no guide but their caprice. Hear the description the German physiologist gives of their evolutions, as detected with the microscope:—"The globules sometimes change their shape, and become double their natural length; but this elongation does not depend on the pressure exercised by the walls of the vessels on them, from these being too narrow. I think that this change of form is due to the inclination of the globule to unite itself to its fellow, whether by virtue of an attractive force, or of their common movement." And elsewhere,—"The globules are always in a state of internal antagonism: at one time they may be looked on as so many particular animal organisms, as infusoria, possessed of something of individuality; at another, as parts of a whole, existing only in relation to the mass, and depending on the general relations of the sanguineous system. This is the reason we see them attract and repel each other, move and be moved, separate from the circulating system, and seek again to re-enter it."

Hence, there can be no doubt that the globules are animals of the order infusoria, maintaining a state of internal antagonism, and borne one against the other by feelings of hostility! Nevertheless, their intercourse is occasionally of an amicable kind: but M. Döllinger must describe their pastimes. "I have seen," says this gentleman, "two globules meet, stop, swing against each other, repel each other alternately, approximate and separate; at length one of the two yielding, takes a determinate course, and returns, followed by the other." It is really fortunate that these little creatures find the means of chasing away ennui in these sports; to be for ever turning and jostling each other would have been by far too monotonous an existence.

With ideas like these on the functions of the globules, Döllinger has made these bodies play a most dramatic part in the phenomenon of inflammation. I might extract some additional passages of his book to show into what extravagant notions the imagination may betray even the best understandings; I shall, however, content myself with the specimens already quoted, and pass to the theory of Kaltenbrunner, one of Döllinger's pupils. Kaltenbrunner, on intercepting the action of the heart in a frog's web, observes an oscillatory movement follow, and continue for a certain time in the capillary canals,—he inquires into the source of this movement. "We must not," he says, "seek for its cause elsewhere than in the blood itself. That fluid is formed of globules; movement is an innate property of those bodies; they originate in movement, exist by movement, and disappear when they lose their mobility." Further on we are informed,—"When the movement of the globules slackens, they immediately lose the accurately defined character of their borders; the latter cease to be at all distinguishable when the globules stand at rest. One is undecided whether it should be said that they disappear because they have ceased to move, or cease to move because they disappear. . . . . . The globules, besides their innate mobility, have also an inclination to move towards a central point, that is, towards the heart. This inclination is perfectly evident in the movement of the lymph, a fluid which has so strong an analogy to the blood. . . . . The lymphatic globules possess this intrinsic mobility as well as those of the blood and of pus."

"The molecules of the blood have a natural tendency to move in the capillaries in such manner as to enter the veins. This phenomenon must be looked on as a very important one in the act of the circulation; but, on examining into this property in healthy organs, it may be objected to this statement that, considering the velocity and regularity with which the blood is propelled into the arteries, to return by the veins without the least interruption, the heart is, to all appearance, the sole cause of the movement. But the harmony which subsists in the state of health between the forces maintaining the circulation in action is the sole cause of this illusion. . . . . The influence of the heart only is perceived, while that of the blood in the capillary vessels remains concealed. To prove the equal share taken by these two forces in the phenomena of the circulation, we have a means offered by nature in a variety of morbid states,—this means is *inflammation.*"

In the explanation of the phenomena of inflammation the same independence of the globules is maintained to exist; nay, more, they are not only said to be independent of that organ, but actually to oppose to it a rival and superior power. A struggle ensues, which Kaltenbrunner thus describes:—"The inclination of the blood to return to the heart undergoes a change in inflammation, of which the nature is unknown. The blood, instead of flowing towards the heart, is then inclined to accumulate at the point of inflammation, which assumes the character of a second heart. The circulation may be now looked on as the result of a conflict between the true and false heart." . . . . . . . . "If the intensity of the inflammation increase, the circulation ceases completely at the point where it attains the maximum of violence, that is, towards the centre; the more intense it is, the greater its extent becomes, and the more the quantity of blood that flows towards the inflamed part augments. At length the animal dies; the force of the heart is annihilated; the vessels of the periphery of the body empty themselves, and the blood ceases to circulate, except in the immediate vicinity of the diseased part. Long after death oscillations continue to be perceptible in the vessels round the inflamed part; whereas the circulation and all motion of the blood have ceased for some time previous to death in those that are healthy." All is over, then, with the animal and his true heart! The false heart has got the upper hand, and, enjoying the victory, pursues its manœuvres in full security, for it no longer need fear interruption on the part of its defunct rival. But the chances of war are capricious; we shall now see the false heart succumb in its turn. "When the inflammation is less violent, the *vis medicatrix* of nature, and that of the heart, gradually recover their energy; the afflux of blood round the inflamed part diminishes, its movements grow regular, hæmostasis is at an end; and thus the equilibrium between the force which maintains the circulation by means of the heart, and that which depends on the capillary vessels, is re-established."

Gentlemen, I am unwilling to push our truly painful examination of this writer's extravagant conceptions any further; if tales such as these were not given to us as exact and well-observed facts, we might perhaps smile at their fantastic oddness; but really, brought forward as they are, they put one's patience to a most severe trial. Of course you do not expect one to set about refuting seriously such a host of absurdities; their simple rehearsal is, in itself, the severest criticism with which it is possible to lash their authors.

So far we have only considered the anatomy of the question; the causes, progress, termination, and treatment of inflammation, should also be submitted to minute examination, and no longer abandoned to the discretion of the nosologists. As for the causes of inflammation, these are so numerous and varied that one cannot comprehend how agencies of so utterly dissimilar a nature should produce the same effect. Let us take, for example, the mucous membrane of the eye; its superficial position and naturally white colour allow the slightest changes in its circulation to be immediately perceived.

An atom of straw gets entangled between the lid and globe of the eye; if it remain there a few moments only, the redness induced in the membrane is transitory, disappearing totally on the removal of the foreign body; if, on the contrary, it remains longer in contact with the conjunctiva, that membrane reddens and becomes painful, and epiphora and an altered condition of its secretion supervene; ophthalmia has set in. Again, divide the fifth pair in the interior of the cranium, on the pars petrosa, and far away from the eye; one of the effects of this section is to destroy the sensibility of the conjunctiva,—the eye becomes as void of feeling as the epidermis. Nevertheless, an ophthalmia quickly follows the division of the nerve. Here, then, we have inflammation preceded in one case by an exaltation, in the other by extinction of tactile sensibility. You will not surely assert that the disease originated in irritation in both instances; it would be too absurd to refer to the same principle such dissimilar causes. It were much better to confess our profound ignorance of the intimate nature of the irregularities thus developed in the capillary circulation.

The causation of the disease in the foregoing instances is, to a certain extent, intelligible; but what one might refuse to believe, if observation had not many and many a time proved the fact, is, that the kind of alimentation to which an animal is submitted exercises a special influence on the circulation of the organ in question. Dogs fed exclusively on gelatine, albumen, or any other proximate principle, are invariably attacked with ophthalmia, which brings on softening and perforation of the cornea and total destruction of vision; and in some cases this ocular affection makes its appearance before the general health is seriously disordered.

Do you wish for another example? You have it in the conjunctival ecchymoses, injection, and puriform secretion, the increased sensibility of the retina, the perversion of the nutrition of the membranes of the eye in our *defibrinised* animals; but if, instead of diminishing the quantity of fibrin in circulation, you simply deprive it of the faculty of coagulating, as, for instance, by the injection of subcarbonate of soda, ophthalmia is then, too, developed. It suffices even to inject a little putrid water into the veins to produce inflammation of both eyes, or of one. But these are not all the causes of ocular inflammation that may be enumerated; far from it: the action of cold air, of damp, external violence, insolation, the reflection of the rays of the sun from white surfaces, the habit of working at very minute objects, and by artificial light, wounds, burns, deviation of the eyelashes, &c. &c., are to be added to the list. Again, we have scrofulous, venereal, blennorrhagic, gouty, rheumatismal, variolous, psoric, morbillous, scarlatinous ophthalmia, &c.; in a word, there is a scarcely a pathological condition which may not lead to the development of that disease. It would be a senseless attempt to endeavour to range, under a common head, so many and so various morbid elements; why, then, designate them by a common epithet?

What is true in the case of the eye, is true in that of every other organ as well. I know not how it has come to pass that persons maintain that every material lesion of our organs originates in inflammation, and this in perverted vitality of the solids. Do not forget that every tissue derives from the blood the materials of its structure; modify the blood, and you at the same time modify the progress of the fluid through the capillaries, and, in consequence, the nutrition of the various parenchymata. You may even, by particular kinds of diet, transform the substance of an organ into a totally different matter; this I have done in the case of the liver. I had remarked, in some experiments on the injection of fatty liquids into the veins, that the tissue of the liver assumed a singular aspect; and had even hazarded a conjecture that by varying the process, one might succeed in making *fatty livers* at will; and, in fact, when I resumed my researches on different sorts of alimentation, I found that animals which had been fed exclusively on butter, or fat, all of them presented, on examination after death, that particular state of the liver known by pathologists under the name of *fatty*. During life no single symptom occurred authorizing any suspicion of the change effected in the structure of the organ; the appetite continued tolerably good; the health apparently satisfactory. You see here the liver of one of these animals; it presents all the characters of fatty degeneration; pale, faded-leaf colour, and friability of tissue; when I plunge a scalpel into its substance, the blade is withdrawn smeared with fat. If you take a slice of this liver, and rub a piece of paper well with it, the character of the combustion of the latter will indicate the presence of fat. I begged of M. Frémy to analyze the fatty liver of several of these animals, and that young chemist ascertained that this condition of the liver is produced by the deposition of a considerable quantity of stearine in the areolæ of its parenchyma. The other constituent principle of fat, namely, oleine, M. Frémy was unable to find. I was desirous to learn whether the fatty liver of the human subject was similarly impregnated, and found here, too, that the chief fatty ingredient was stearine. This result appears to me really interesting, and one possibly calculated to throw some light on the etiology of this disease of the liver.

These facts show the possibility of our being able to substitute milder means for the barbarous practices now employed to render the liver of certain fowl fatty; why might not this end be as well attained by changing the nature of their food, as by putting out their eyes, confining them in the damp, deforming their chests, and cramming them almost to suffocation with vegetables?

Here is a dog which has for the last three weeks been fed on unpurified beef fat. He is weak, thin, and dejected; I have no doubt that the liver is in a commencing state of fatty degeneration. You recognise in this animal a proof of what I told you just now respecting the influence of regimen on the production of ophthalmia; its eyes are red, and the lids coated with puriform matter. I beg you will notice the kind of fatty coating that agglutinates together the animal's hairs, and gives them a shining aspect. Might we not hazard the supposition that the oleine has escaped by the cutaneous exhalation, while the stearine has been deposited in the liver; it would be certainly worth while to test the correctness of this conjecture by chemical analysis.

I could not adduce a more striking example than this, to demonstrate the immense importance of alimentation in respect of the nutrition and diseases of our organs. Observe the harmony that subsists between the blood and the vessels containing it. So long as that fluid retains its normal characters, it traverses the capillaries of the liver freely; the moment it grows too viscid, it stagnates and allows some of its materials to pass by infiltration into the parenchyma of the organ. I do not myself know any signs—nor do I believe such are known by any one else—whereby the existence of a fatty condition of the liver may be diagnosticated in the living subject: except in certain cases of phthisis it is even impossible to suspect its presence. But suppose it ascertained that the liver is thus affected, what mode of treatment should we advise? Purgatives, to *stimulate the biliary secretion and disgorge the liver;* leeches to the anus, *to unload the mesenteric veins;* moxas and issues to the right side of the abdomen, to *displace the irritation;* venesection, to *lower the inflammatory state;* and many similar agents would, no doubt, be employed by the routine practitioner. For my part, if I had to combat an affection of the kind, I should commence by inquiring into the previous regimen of the patient, and ascertaining if he had not made excessive use of fatty substances; that is, of butter, fat, and oil. If such were the case, beyond a doubt the first thing to be done would be to change the patient's regimen; the liver might, possibly, then recover its normal structure. We know that when individuals use sorrel to excess, and, in consequence, void oxalate of lime gravel in their urine, this morbid condition of the renal secretion may be totally removed.

The inference from all I have now said is, that the properties of the blood cannot be modified without the occurrence of pathological phenomena in the capillary circulation. What we see occur in the conjunctiva permits us to judge of what takes place in deep seated organs. Far from inquiring into the cause of these disorders, people are generally contented with referring them to favourite theories, and, with a word which is essentially meaningless, fancy that they express most important facts. If the circulation be suddenly disordered, we have an *acute* inflammation; if the progress of things be slower, the inflammation is *chronic;* if any tissue be found in a disorganised state, when no morbid process was previously suspected, still there has been inflammation; but here it has been *latent*. Every thing is thus easily explained, especially whatever is, in truth, inexplicable.

If our acquaintance with the manner in which inflammation originates be imperfect, our notions respecting its various modes of termination are no less so. Here, too, we find nothing but routine classifications, based on the grossest of the phenomena presented by the diseased tissues. According to authors, the various modes of termination of inflammation are reducible to six categories: those of *delitescence*, *metastasis*, *resolution*, *suppuration*, *induration*, and *gangrene*. An inflammation being given then, the only difficulty consists in fixing to which of these categories it belongs. This would, no doubt, be simple and most ingenious, if each of these groups were founded on a reasonable theory of the phenomena referrible to it; but such is not the case: words we may have in abundance; correct ideas few or none.

By *delitescence* is understood the sudden disappearance of inflammation. Why this prompt cessation of the morbid symptoms? Because the circulation, which was momentarily disturbed, resumed its natural course before considerable obstruction and extravasation had time to form. You have an example of this in what occurs when you apply a burning liquid to your hand; it reddens; more blood than natural flows to it; there is now *irritation;* plunge it into ice-cold water, the quantity of blood rushing to the part is diminished; the redness decreases; you have arrested the *inflammation* in the outset. I have used the expressions irritation and inflammation to make you feel how unfit these words are in such a case, inasmuch as they turn attention from the chief point, the influence exercised by temperature on the progression of the blood in the vessels. For the just comprehension of these phenomena, I beg to refer you to the experiments on cold and heat with the hæmodynamometer.

By *metastasis* is meant the sudden and spontaneous removal of inflammation from the part it occupies to some more or less distant quarter. I shall not trouble you with all the hypotheses which have been imagined to describe the route taken by the inflammatory elements in such cases. Some make it travel with the blood; others have supposed it to be summoned by the sympathies; others have been reduced to making it jump from one place to another by virtue of an unknown power, emanating from the vital properties. There is a deal of vagueness connected with the meaning of this word metastasis; the solution of the problem is reserved for experimental researches.

*Resolution* is the gradual disappearance of inflammation, and constitutes its most favourable termination. The disease in this case goes through all its periods *gradatim;* the pain diminishes, the swelling disappears, the parts return insensibly to their normal condition, and the free exercise of their functions. How do the obstructed vessels recover their lost properties? I believe that their recovery depends on the modifications undergone by the blood accumulated in the part. This blood at first became solid by the separation and infiltration of its liquid portion through the pores or ruptured openings of the vascular tunics; at a later period the coagulated fibrin liquefies, becomes fluid again, and is carried along by the blood, propelled by the heart's action. Once the capillaries are rendered free in their interior, the extravasated materials re-enter the circulation, and the swelling disappears. The increased heat of the inflamed parts explains very clearly these chemical changes undergone by the blood accidentally arrested in its course. Observe what happens in vast ecchymoses produced by violent contusions: the blood remains in the fluid state for a certain time; its aqueous part then passes by infiltration into the circumjacent tissues; a solid coagulum occupies the centre of the injured part; this clot gradually softens, breaks down into small pieces, which dissolve in the serosity, and when rendered perfectly fluid are wholly removed by absorption. By the same mechanism, as it appears to me, the resolution of inflammation may be explained; nevertheless, these views require substantiation with the microscope.

When the materials of the blood, arrested in the vessels, or extravasated from them, are softened, they do not always re-enter the circulation; too prolonged a sojurn in the inflamed part disorganises the structure of the tissues, and life, momentarily suspended therein, threatens to cease almost completely. The tumour gradually loses its hardness, its centre *points*, and there fluctuation is perceptible, while elsewhere it is hard and tolerably firm; in other words, the affection terminates in *suppuration.* Pulsative pain, fluctuation, and attenuation of the skin, announce the formation of pus, when the inflammation is superficial; but it is more difficult to assure oneself of its presence when the disorder is deeply seated in the limb; we are then obliged to have recourse to rational signs, with which I have nothing at present to do. You perceive that the essential difference between the terminations by resolution and by suppuration consists in the fact, that in the former the molecules of blood are absorbed after having softened; whereas, in the latter, they undergo further modification, are completely transformed, and expelled from the system.

Authors are not agreed as to the mechanism of suppuration, and as to the substances which produce pus: the question does not, however, appear to me unanswerable. We have seen the principal materials of the blood become extravasated, and so constitute inflammatory engorgement; in proportion as they liquefy they combine, intimately, with the tissues in which they are effused, so that pus is formed at once from the solids and liquids. What proves that it is formed in the diseased part itself is, that it assumes particular characters, according to the organ or tissues in which it is found: thin and grayish in the bones; opaque and caseiform in the cellular membrane; flocculent in the serous, and greenish and thready in the mucous membranes; reddish in the liver; yellowish-gray in the muscles; pus presents special characteristics wherever it is examined. One of the gentlemen present, Dr. Gluge, is able to distinguish from each other the different species of pus by the simple inspection of their globules. I have been among the witnesses of a variety of tests to which his powers have been submitted, and, in every instance, he has been successful. I brought pus from the hospital which had been collected in the lung, the pleura, the peritoneum, and the cellular tissue, and he invariably announced its origin with perfect correctness. I recollected even having endeavoured to entrap him by presenting him with some artificial pus of my own making, as though taken from one of my patients, but he was not to be deceived. I have therefore, not the least doubt that the globules of the various species of pus may be recognised by certain physical characters. This important fact is another to be added to the list of experimental discoveries.

When the inflammatory engorgement remains stationary, when the hardened condition of the tissues augments, while the other inflammatory phenomena disappears, the disease is said to terminate by *induration.* This mode of termination is proper to glandular organs, and generally succeeds a slow, obscure inflammatory action. The modifications undergone by the indurated parts are not yet precisely understood; all we know is, that the extravasated matters do not re-enter the circulation, and that they become organized, and soon form an integral part of the tissues. By what series of phenomena does inflammation of the testicle effect the transformation of the testicle into a scirrhous mass? How comes it that encephaloid of that organ often appears to follow simple disordered condition of the progression of the blood? There can be little doubt that the nature of the materials effused gives a particular character to the manner in which the disease terminates. According as these remain solid or liquefy, the consistence of the part undergoes changes which would furnish most interesting matter for analysis, but which writers have been contented to designate by different epithets without seeking their cause.

On the termination by *gangrene* I have already spoken to you at sufficient length.

It remains for me to discuss the treatment of inflammation; but this is so vast a subject, gentlemen, and the indications it embraces are of such an import, that it would be impossible for me even to glance at its chief points in the few minutes I have still at my disposal. Let me state, however, that if the phenomena of inflammation cannot be referred to one origin, the same is even more emphatically true of its treatment. What is to be understood by the strange denomination of antiphlogistic? Will broths and mucilaginous liquids restore the blood its coagulability, and prevent its extravasation through the coats of its vessels? Are leeches the sole means of removing the *thorn* that stimulates the inflamed part? It is rational to attack the blood, as the movement of that fluid is disordered; but it is its composition much more than its volume, that requires to be modified. What is well fitted for one inflammation, would be injudiciously employed for another. In one case the action of the organs needs stimulation,—in another depression; in one patient the blood ceases to circulate, because it is too fluid; in another, because it is too viscous; in a word each inflammation calls for its particular therapeutical measurer.—*Lancet.*

---

*Remarks on the use of Colchicum and Lyttæ Topically in Rheumatism and Vesical Paralysis.* By T. Laycock of York.—Some theoretical speculations led me to try the following liniment in rheumatism:—

℞ Tr. Rad. Colch.; Tr. Camph. aa. partes æquales. M.

The patient who used this was a tall groom (Richard Bould,) under the care of Dr. Belcombe, subject to rheumatic attacks, and who at the time was unable to lift his arm, on account of rheumatism of the deltoid muscle. I was agreeably surprised to find that, after the third application, and within twelve hours after the first, he was able to raise his arm freely to his head. The relief was, however, only temporary, but the application was used with equal success so often as the pain recurred. The patient was subsequently attacked by small-pox (after vaccination,) and nothing was heard of the rheumatic pains until he was convalescent, when they attacked his hip. He reminded me of the liniment, and one trial removed the pain. I now prescribed it for two or three out-patients, and these derived benefit. I then omitted the tincture of camphor, and I now find the groom is relieved with equal celerity and certainty by the tincture of colchicum-root alone. Relief so constantly follows its application in his case, that I cannot doubt its utility. When the loins are affected he cannot turn in bed unless the tincture be previously used. He rubs one or two teaspoonsful on the part affected. I have found it equally successful in another case, in which the deltoid muscle was affected.

The only notice I can find of this method of using colchicum, is in the "Dictionnaire de Mat. Medic." of Merat and De Lens, ii. 361. A Dr. Gumpert is there quoted (from Rev. Méd. i. 140,) as having used the tincture of seeds of colchicum as a local application in gout and rheumatism very successfully. The particular instance of a clergyman is mentioned, who was confined to his bed for a month or six weeks with the latter, and who was able to leave it on the fifth day after friction with the tincture of the seeds. From theoretical considerations, which I need not detail, I think it would be found a useful application in gout as well as rheumatism. Those who have corns, which are painful during atmospheric changes, will probably find the twinges of those delicate pedal barometers alleviated by the topical use of some preparation of colchicum. *Bursal* rheumatism will, of course, be most relieved by its use.

*Lyttæ in vesical paralysis.*—I believe it is well known that the tincture and powder of the *melöe vesicatoria*, or cantharis, is very useful in atony or paralysis of the bladder, especially of hysterical and aged people. I have found, however, that an *emplastrum lyttæ* applied to the loins is equally efficacious, and much more manageable. A female, confined to bed in the last stages of laryngeal phthisis, could not pass urine without raising herself upon her knees. She was at last too weak for the effort, and it became a question how the difficulty could be surmounted. I recommended an emplastrum lyttæ to be applied to the loins or sacrum, until she felt able to empty the bladder in the recumbent posture. In half an hour after the application she succeeded. She lived for three or four weeks subsequently, and the plaster was in almost daily use until she died. In most instances from one to two hours elapse before the desired effect is produced; in hysterical retention about the latter period. The plaster is useful in other cases. A man came to the hospital with a catheter in his bladder; he had not made water without it for three weeks. It was removed, and an emplastrum lyttæ applied to the sacrum for three or four hours; he never wanted the catheter again, and went away in a week quite well. I am not aware that this method of using the fly is mentioned by authors.

Dr. Simpson (physician to the hospital) uses a belladonna plaster over the region of the heart, to quiet violent palpitation; I have found it very successful, especially in nervous palpitation. The belladonna plaster will also relieve irritable bladder, and neuralgia or irritability of the rectum. The plaster should be made with the pure extract spread on lint or leather, and applied moist to the sacrum or perineum. I think an opiate plaster made with powdered opium and soap cerate, is more efficacious than the belladonna, at least in irritable bladder; it will sometimes enable a person to rest undisturbed during a whole night.

*Lon. Med. Gaz.*

---

*Clinical Observations on Disease of the Vertebræ.* By Sir B. Brodie.—There is a joint between every two vertebral bodies, with the intervention only of a piece of cartilage between them. If, in a case of diseased vertebræ, you dissect the parts in the early stage of the complaint, you will sometimes find the bones more vascular than natural. After this a cheesy deposite takes place, and the vuscularity of the part diminishes. At other times you will find that the vertebræ are abnormally light, soft, and spongy in texture, admitting of being easily cut through with a knife; and, again, in other cases, you may find them unusually hard and heavy in texture, as bones frequently are in chronic inflammation, before ulceration sets in. Diseases of the vertebræ sometimes commence in the intervertebral substance. In the healthy state there is, in the centre of the cartilage, an elastic, soft, gelatinous substance. This sometimes becomes brown and brittle, and seems divided into fragmentary lamellæ, and loses its connecting adhesion above and below. In this manner caries will sometimes commence in several parts of the vertebral column at once. In other cases the ulceration begins at the anterior or lateral surfaces of the vertebræ, but most frequently its first effects are seen at that part where the vertebral bodies are connected to the cartilage above and below. When ulceration once commences, it spreads very rapidly, and may continue for some time before suppuration comes on. This may occur when as yet, there is but little destruction of the bones from ulceration, or the contrary; or the vascularity of the bones may diminish and they may die, but not exfoliate to any great extent. Ulceration may go on in the bodies of the vertebræ, as in other joints, for a long time without suppuration coming on; but, sooner or later, abscesses will form on the surface of the carious bone.

Sometimes suppuration begins with but little destruction of the vertebral bodies, and but a very small quantity of pus may be secreted. Sometimes, also, suppuration will not commence until ulceration has proceeded to a very great length. When the bodies of the vertebræ die they exfoliate as in scrofulous cases, their vascularity becoming diminished, and the sequestrum is thrown off, but not to any very great extent. The vertebræ are some times extensively implicated, and many surfaces of bone are affected, whilst, in other cases, the reverse of this obtains. When an abscess forms it points sooner or later to the surface, according to its situation. Sometimes, however, it makes its way inwards into the cavity of the theca vertebralis. I remember one case in which the cervical vertebræ were affected, and the whole cavity of the theca was filled with pus.

Caries sometimes occurs in the joints, between the articulating processes, and this is more frequently to be met with than is generally supposed. This most frequently occurs in the cervical vertebræ, and the destruction of parts is, in these cases, greater than where the disease is confined originally to the bodies of the vertebræ.

Where caries affects the bodies of the vertebræ you do not notice, at first, any alteration in the figure of the spine; and this alteration, when it occurs, is marked by an angular curve, greater or lesser, according to the situation of the original disease. In the lumbar vertebræ, the disease may go on for a long time before any alteration is observed in the shape of the spinal column, because their spinous processes are short and stand directly out. The same may also be said of the cervical vertebræ. But, in the dorsal vertebræ, the bodies of the bones are small, compared to those of the loins; their spinous processes are long and point downwards.

Angular cuvature, then, only occurs in the advanced stages of carious disease of the vertebræ. Sometimes it shows itself suddenly, in course of a month, perhaps, in cases where matter forms suddenly, and as suddenly discharges itself. Angular curvature differs in different cases; sometimes there is a double angle, one below the other. If the curvature be slight there is but little alteration of position in the internal viscera; if the curvature be great, the course of the aorta is altered, and I have dissected cases after death in which the aorta made two or three turns. Sometimes, in these cases, the sternum and ribs project more than is natural, the heart appears displaced, and the lungs seem compressed and diminished in size.

I shall now give you a general account of this disease of the vertebræ as it is developed in its symptoms. The pain is sometimes very obscure and trifling in the early stage, and it may gradually increase to very great severity, or it may remain very trifling in degree throughout the entire case; or it may, on the contrary, be very severe from the first day to the last. I have known cases in which the curvature has been very great and the patient has suffered no pain whatever; whilst I have known others in which the curvature was much less, and the slightest motion gave the patient intense pain, and pressure caused very great agony. Here, then, are two extreme cases; but between these are many degrees of variation and change. In scrofulous caries of the spinal bones there is, generally, but little pain suffered, whilst in simple inflammation, followed by ulceration, the pain is generally very severe. This disease of the vertebræ begins sometimes very insidiously, and can be traced to no original source or cause. It will sometimes follow an attack of fever, and caries, with all its symptoms, becomes soon set up. There are other diseases of bones which frequently show themselves after an attack of fever. Sometimes this occurs in persons of truly scrofulous habit; sometimes in those who possess the healthiest constitutions. Pain comes on first, and suppuration and abscess soon follow. In some cases, however, a long time elapses before any thing in the shape of a spinal column occurs, or abscess presents itself. I know of a case in which abscess only showed itself ten years after the erruption of the original disease; and I know of another in which the disease had existed twenty-one years before any abscess presented. An abscess, therefore, may be pent up for a very long time. It is very strange, however, that it may exist for this length of time without the constitution suffering from its irritation.

Well, then, either sooner or later, the abscess bursts. When this occurs in young persons they may recover, the cavity may become filled up, and anchylosis may take place between the vertebræ. Generally, however, the reverse of this presents itself, and the patient dies. Hectic fever shows itself; the lungs become affected, and the patient dies from some internal disease supervening upon the fever. The children of the upper classes of life may, and do sometimes recover from the disease.

Disease of the bones of the spine may irritate the spinal chord, or the cauda equina, and cause irritation of the parts below which they supply with nervous influence. These symptoms differ, of course, according to what part of the bone the caries is situated in.

If the caries be situated in the upper cervical vertebiæ, there is pain in the back of the neck when no motion takes place, and stiffness; and such a case may be simply mistaken for one of stiff neck. There is pain extending up to the head, and this may last for a long time before angular curvature shows itself, because the spinous processes of the cervical vertebræ are very short. The symptoms which then ensue are numbness and paralysis, with loss of the use of the arms, pains in the shoulders and arms, which may be severe or not. This is followed by paralysis extending from muscle to muscle. If the disease goes on, these symptoms extend to the lower limbs, and they become paralysed, and the patient cannot move at all. The abdomen becomes affected, the bowels become torpid, and no purgatives will act upon them unless they be combined with ammonia. Some time will frequently elapse before abscess forms in the neck, which generally occurs at its inferior and lateral part. You should be careful in detecting this disease in its early stage, and you may know it by the accompanying pain and stiffness. If you put your hand on the patient's head and press it down firmly, it will cause very great pain, which simple stiff neck will not do.

When caries occurs in the dorsal vertebræ, there may be pain or not at the actual seat of the disease, or the pain may be in the loins, or referred, in the first instance, only to the hip, which might lead an inexperienced practitioner to imagine the disease was situated there. As the disease goes on, then, in the dorsal vertebræ the pain extends; the chest and abdomen, the bowels become torpid, and there is frequent sense of constriction across the epigastric region, with tightness of the chest and dyspnœa. The lower limbs become paralysed, and the patient trips in walking. The muscles are affected by spasmodic twitchings and convulsive movements. The bladder becomes paralysed, and the urine is with difficulty voided, whilst the spine becomes distorted, more evidently so in the neck than elsewhere, from the spinous processes of the cervical vertebræ pointing downwards. As the disease progresses the angular curvature increases; an abscess forms at the anterior and lateral surface of the spinal column, which is bound down by a thick capsule of lymph, preventing it frequently from coming forward in the spot in which it forms, and frequently causing it to descend along the line of the psoas muscle, and present in the groin.

If caries occurs in the lumbar vertebræ the abscess may be felt in the belly, in front of the kidneys, where it may lodge for a long time, and thence descend along the psoas muscle in the groin, pass thence through the sacro-sciatic notch, following the course of the sciatic nerve, and coming out at the back of the thigh; or it may burst at the back of the abdomen; or I have known cases in which it has come forward in the chest. The pain in the loins may be at one side only, or it may extend across to both, and it may be slight or severe in suffering, according to the condition of the bone, whether it be hard or soft from scrofulous diseases or chronic ulceration. In some cases the pain is severely aggravated by the slightest motion of the lower limbs, and this may continue for three or four years. I knew one in which this state of disease continued for ten years. After this abscess appears. Sometimes there is paralysis of the parts below; sometimes not; and sometimes there is angular curvature, and sometimes not. In these cases the abscess will present itself at the anterior superior spinous process of the ilium; and sometimes by the side of the sacro-lumbalis, or it may burst into the rectum or the scrotum, or it may present at one place and shift thence to another. When it presents itself in the groin it is small, but very soon increases in size. An abscess may be present but cause no constitutional disturbance; or there may be frequent rigors, night sweats, and hectic fever.

Within the spinal canal of the lumbar vertebræ there is no spinal marrow, but only the cauda equina, each with its separate neurilema. The bodies of the lumbar vertebræ are larger, and it therefore takes a longer time for an abscess to eat its way out by ulceration. It is, therefore, a longer period before paralysis occurs, and a greater degree of ulceration is required to produce angular curvature; and when this does occur, the projection outwards is not so well marked, from the spinous process being shorter.

You may safely diagnosticate between this affection of the lumbar vertebræ, and others in which the bones and muscles of the loins are affected. Common lumbago comes on suddenly, with pain and incapability of motion, which, after a time, go off. Inflammation of the lower part of the spinal chord and the cauda equina produce pain with effusion of lymph around the spinal marrow; the sudden and violent pain in the loins resembles lumbago somewhat; but you may distinguish it by the pain not being increased on motion, and if you cup and bleed, and give mercury, the pain and paralysis subside. Pain from affection of the kidneys generally occurs in one loin only, with consequent irritation of the bladder, with albumen and pus in the urine, which latter symptoms will sometimes render the diagnosis difficult. I knew of a case of albuminous urine, occurring in a case of diseased spine, mistaken for disease of the kidney; but whether disease of the spine will extend to the kidney, my experience does not enable me to determine.

*Ibid.*

---

*A Clinical Lecture on Syphilis.* By Professor Graves.—In one of my first lectures I stated that, notwithstanding the host of facts bearing on the question of the non-mercurial treatment of primary and secondary syphilis, there is still much difference of opinion amongst men of the highest rank in the profession. One good has resulted from the statements put forward by the army medical practitioners, namely, that mercury is no longer abused in the empiricle and barbarous manner followed by our predecessors. Few, if any, at the present day, will be found to enter upon long and exhausting courses of mercury, for slight chancers or sores, in persons of delicate or scrofulous constitutions; and I believe the opinion is growing stronger and more general every day, that when primary symptoms occur, although mercury be omitted, or merely used as an alterative, the disease may be successfully treated. Let me, however, be understood in this matter. I make this statement in reference to those cases only, in whice the disease is treated from the commencement, and not allowed to go on unchecked for days or even weeks. I have already brought forward evidence to prove, that when genuine chancre is treated properly from the beginning, it may be cured without mercury. There must have been several cases of true chancre among Dr. Roe's patients, and yet of the entire number there was only a single case of secondary venereal, and that in a patient broken down in health, and labouring under bubo for a considerable time before admission.

But you will ask—Is it possible to cure secondary symptoms without mercury? If you are to believe some authors, you cannot. According to their views of the case, a patient labouring under secondary symptoms, if treated without mercury, may get well for a while, but the disease will return again and again until it breaks up his health. All I can say on the point in question is this, that I have seen several cases which were pronounced secondary syphilis get completely well without mercury. About ten or twelve years ago, there was a case of secondary syphilis in this hospital, which I treated without mercury. It was a case of well-marked papular disease, which had made its appearance about six weeks after the primary sore; and, to remove all doubts on the subject, I showed the man to the late Mr. Hewson—a gentleman justly esteemed for his accurate and extensive knowledge of the venereal disease. He pronounced it at once a case of true syphilis, and added that it could not be cured without mercury. As there was no urgent reason for the exhibition of mercury, I thought the matter worthy of experiment, and treated the man with purgatives and antimonials, followed by vegetable alteratives and nitric acid. I did so, and succeeded in effecting a perfect cure. I kept the man afterwards under surveillance, to see if a relapse would occur. He never had a return of the disease, and Mr. Hewson was quite struck with the result, as he had no conception that the patient could be cured without mercury. Indeed, this was the general opinion, the other surgeons of the Meath Hospital having arrived at the same conclusion. The case made a very strong impression on my mind, and, connected with others having a similar result, has convinced me that there is some truth in the statements of those authors who say that syphilis can be cured without the mineral. On the other hand, I must confess that there are some cases which answer the description given by Mr. Colles, and which cannot be cured without bringing the patient under the influence of mercury. Thus, a very fine healthy young man, whom I attended some years ago, put himself under my care for chancre, after having neglected the disease for three weeks or more. Now, when a case of this kind, which has been allowed to run on unchecked, comes before you, you should not be too sanguine, or think that your patient will be perfectly safe under the non-mercurial treatment; for where chancres are neglected, secondary symptoms are very apt to occur. I treated him with purgatives, antimonials, rest, and low diet. He had no buboes, and got quickly well; but about five or six weeks afterwards, he was seized with symptoms of fever, accompanied by acute pains of the joints, and two days afterwards got venereal eruption and sore throat. He had, in fact, all the symptoms of venereal exanthematous fever, and his skin became covered with blotches—the character of which could not be mistaken.

They were neither papulæ, pustules, nor tubercles, but true venereal blotches, terminating in scaly scurf. I gave him tartar emetic, followed by vegetable alteratives, and he got better. He continued well for about a fortnight or three weeks, and then another eruption broke out, attended with pains and fever, as before. The non-mercnrial plan was tried again, and was again followed by the same apparent success; the eruption faded, and his throat got better. He then took lodgings in the country, for the benefit of change of air, but while there, was attacked a third time, more severely than before. He had fever, eruption, and sore throat, and, in addition to these, periostitis and nodes; he was also becoming weak and emaciated, Under these circumstances, I prescribed calomel and mercurial ointment, until his mouth became sore. His symptoms all gradually disappeared, and he has had no return of the disease. In this gentleman the greatest attention was paid to diet, confinement to the house, and every circumstance which could favour the success of the non-mercurial plan. The patient's constitution was excellent, and free from any scrofulous taint, and the syphilitic poison seemed to be rapidly undermining his strength, and the disease acquired fresh force from time instead of growing less violent; in fact, its progress was so alarming, that mercury could be no longer with safety withheld. A very moderate course of mercury, managed so as to keep his mouth tender for six weeks, thoroughly and permanently cured him.

Now, to what conclusion does all this lead? simply to this, and I believe it is the conclusion to which all rational men have come, that although there are many cases of syphilis, which can be cured without mercury, there are others in which its employment is indispensable.

In the two cases, which I have just related, the results were very dissimilar. In the first, a case which had been pronounced distinctly venereal by some of our most distinguished surgeons, and not to be cured without mercury, the non-mercurial treatment proved quite efficacious; the man was readily cured, and had no return of his disease. The other case, which you would have regarded as most favourably circumstanced for getting well without mercury, had quite an opposite result; the disease returned again and again, and did not yield completely until the system had been brought under the mercurial influence. Hence you perceive the necessity of avoiding extreme opinions, or coming to any general conclusions as to the treatment of syphilis.

The inferences which my experience has led me to draw on this subject, are, that many cases of syphilis—indeed a great majority of cases of primary sores—may be cured without mercury, if treated at once and properly.

After chancres have existed for some time, the chances of secondary symptoms are greatly increased, and mercury in such cases will be often required; but it should be used with caution, and moderately. Were I to speak for myself, I would say, that, as a general rule, I prefer the non-mercurial plan in the treatment of primary chancres, particularly if seen at the commencement, and where they appear in persons of a delicate and scrofulous habit. I think, at least you will not be wrong in giving many cases of chancre a trial, and see whether you can cure them without mercury. If secondary symptoms appear, you have still a resource in mercury; the patient's constitution is unimpaired, and the disease is still amenable to treatment. If you treat your patient properly, he has many chances in his favour; and if he gets secondary symptoms, mercury will still act favourably on his system. The rational practitioner is neither a mercurialist nor a non-mercurialist; he acts according to the state and peculiar exigencies of each case, and selects his plan of treatment according to the form, condition, and duration of the disease, as well as the constitution of the patient. If the chancres be of a mild, and what may be termed indolent character, the application of nitrate of silver at an early period, combined with rest, low diet, aperients, and, if necessary, vegetable alteratives, will complete the cure. If attended with inflammatory symptoms, a vigorous adoption of the antiphlogistic plan will be indispensable, and the use of caustic applications must be deferred until the symptoms of inflammatory action are abated.

Whenever you get a chancre, in its commencing period to treat, try the antiphlogistic and non-mercurial plans, and, if your patient improves, persevere; but, if there be no amendment, you may have recourse to the cautious exhibition of mercury. I say cautious, for in some constitutions you cannot be too careful in the administration of this remedy. The consequences which have followed from the injudicious use of mercury have been often and strongly depicted, but not in colours too strong for truth; the lamentable results which have attended its abuse, rank among the greatest opprobria of medicine.

In Johnson's General History of Pyrates—a most curious book, published in 1725, and from which Sir Walter Scott has borrowed some of his best traits of nautical character—we find a passage proving that the abuses of mercury were great at that period, and that even then, facts were not wanting to show that this mineral was not indispensably necessary for the cure of syphilis. In the following passage I have preserved the spelling of the original. Talking of the Brazils, our author remarks,—"The generallity of both sexes are touched with venereal taints, without so much as one surgeon among them, or any one skilled in Physick to cure or palliate the progressive mischief. The only person pretending that way is an Irish *Father* or Priest, whose knowledge is all comprehended in the virtues of two or three simples, and those, with the salubrity of the air and temperance, is what they depend upon for subduing the worst of malignity; and it may not be unworthy to notice, that though few are exempted from the misfortune of a running eruption or the like, yet I could hear of none precipitated into those deplorable circumstances we see common in unskilful mercurial processes."

Who can read, without shuddering, the long detail of misery inflicted on unfortunate venereal patients in the time of our predecessors? the exhausting salivations—the inveterate nodes—the frightful caries and sloughing—the emaciation—the hectic—the rapid or lingering, but ever fatal phthisis. Hundreds of victims, whose slight primary symptoms might have been successfully treated without a single grain of mercury, have had their constitutions gradually broken down, until at length scrofula became fully developed, and was quickly followed by its attendant, tubercular consumption.

Thanks to the exertions and labours of the army surgeons, we no longer behold the same indiscriminate exhibition of mercury, or the same wicked tampering with human life. The evils which have flowed from the abuse of mercury are greatly diminished, but still not sufficiently exploded from British practice. Notwithstanding all that has been said and done, a good deal still remains to be accomplished, before the treatment of syphilis can be said to be placed on a solid and rational basis. I am not among those who contend that you should never use mercury. On the contrary, I think there are cases in which you can employ it to great advantage—in fact, where its employment is indispensable. But I would have you always act with caution. In treating cases of primary or secondary symptoms, which have existed for some time, and where the patient has been taking mercury, it is hard to unravel the perplexities which surround the case, and ascertain whether the mercury has been properly administered or not.

Where a patient labouring under syphilis has been salivated without being improved, one of two things must be inferred—either that the mineral has had no effect on the disease, or that it has had an injurious effect on the constitution. The great point to arrive at in the treatment of syphilis, is to make the mercury act on the disease, and not on the constitution. This I have often endeavoured to impress on my class. I will venture to say, that I would engage to give a patient labouring under primary symptoms any quantity of mercury, without producing a favourable effect on his disease, or doing him any good: I would engage to salivate a man affected with sore throat, and yet leave him as bad, or even worse, than ever. I have witnessed this occurrence over and over again, and have laid it down to myself as a proposition—that venereal may be treated with mercury, to the fullest extent, without being cured.

Syphilis and mercury are not like two opposite forces—not like an acid and an alkali—so that by putting them together you are sure to neutralize them. No. It is a melancholy fact, but true, that the constitution may be impregnated with both at the same time. Some time ago, a gentleman's coachman was admitted into Sir Patrick Dun's Hospital. He got primary symptoms for which he took mercury; but being of active habits, and unwilling to quit his employment, he remained with his master, whom he was frequently obliged to attend at night. In this way, he was often exposed to wet and cold, and used to take whiskey, with a view of protecting himself. The consequence was, that eight weeks afterwards, he came into Sir P. Dun's Hospital, with his mouth sore and fully salivated, but labouring under bad sore throat, and an extensive eruption. In adverting to his case before the class, I said, "This appears to be a very bad specimen of the mercurial treatment, but you are not to conclude from what you see that mercury will not cure the disease. We will keep him in hospital; give him mild aperients, light nutritious diet, and sarsaparilla; and when we have removed the bad effects of mercury on his constitution, we will proceed to administer it again, but in such a way as to act on the disease, and not on his general health." About three or four weeks afterwards, the man was so much improved, that we were able to put him again under a mild course of mercury, and succeeded in eradicating every symptom of disease. Although a patient has got worse under the use of mercury, you should not conclude that it is incapable of curing the disease: it may have been administered improperly; and under such circumstances, I tell you again, no good can be be expected from it. In such cases the morbid action of mercury must be allowed to pass off completely before we have recourse to the mineral again; and if this be done with circumspection and care, the best and most favourable results may be expected. I agree perfectly with the judicious observations put forward on this subject by Dr. Lendrick, and I would strongly recommend every gentleman present to read his excellent paper, published in the 32d number of the Dublin Medical Journal. As in many acute diseases, particularly those of the class Exanthemata, so in syphilis you may have great variety in the symptoms, some of them will be faintly shadowed out, or altogether absent; while others may manifest a remarkable prominence. In measles you may have the eruption without the catarrhal symptoms; in scarlatina, the sore throat without the eruption, or, what is still more curious, the desquamation and dropsy without any apparent preceding symptoms. So also in syphilis, in which you may have chancre without bubo, sore throat without eruption, or periostitis without any well-marked appearance of the symptoms which usually precede it in the order of time. You are not to expect that the disease will always appear in the form laid down by the great John Hunter, or that the symptoms will pursue the precise order marked out by him. As in an acute disease, where not merely a single symptom, but even whole groups of symptoms, may be absent; so in many forms of chronic disease, some of the characteristic marks will be occasionally wanting. There is much variety in forms, intensity, complexion, and duration of chronic diseases, and particularly with regard to those which arise from animal poisons. Scarlatina, typhus, measles and small-pox, produce very different impressions on different constitutions, operating on some mildly and favourably, on others with extreme intensity. The same variety is seen in the constitutional symptoms produced by syphilis; in some they are slight and chronic, in others acute and violent. In fact, syphilis is so variable a disease, that every reflecting and experienced observer will be led to the conclusion, that it must require a mixed and varied treatment, and that its treatment cannot be based on any general code of laws, as laid down by mercurialists or non-mercurialists. By acting in this way, you will avoid both extremes, and pursue a wiser and a better course.

There is another point to which I shall direct your attention before I conclude. It is of great importance in the treatment of venereal affections, to bear in mind, that there are other poisons capable of producing an eruption similar to the syphilitic.

In a lecture published last year, I endeavoured to show, that in some deranged states of the constitution, the human body is capable of generating an animal poison within itself, one of the characters of which is a more or less general cutaneous eruption. I have also shown that deranged local action of a part of the body may be followed by inflammation and the formation of matter capable of infecting the whole constitution. I have more than once, while going round the wards, been struck with the appearance of a sore of this description, and on stripping the patient, have found some of Mr. Colles's pustules on the skin.

Some time ago, a young man came into this hospital with gonorrhœa and phymosis: he was unable to draw back the prepuce, and the consequence was, that the extensively ulcerated glans lay constantly bathed in gonorrhœal matter. Shortly after admission, his skin became covered with an extensive papular or papulo-pustulo eruption, which was looked upon by many as true venereal. He also became emaciated, and sore throat, very closely resembling syphilitic sore throat, made its appearance. The prepuce having been divided, he was treated with small doses of arsenic, mild nutritious diet, rest, and lotions of sulphate of zinc, and recovered completely. A case still more curious occurred some time since. A gentleman, one of the pupils, cut his finger while dissecting. The wound was followed, some time after, by a suppurating tumour, resembling a whitlow, which lasted for a long time, and finally generated a poison, which produced sore throat and a cutaneous eruption, the latter of such an obstinate character, that, after trying many remedies, he was obliged to have recourse to mercury. These facts, coupled with others of a similar tendency, show that venereal symptoms present a considerable variety as to their number, order, form, duration, and curability by mercury, consequently it often becomes a matter of difficulty to distinguish the true nature of the disease, and separate it from other influences, by which it may be modified. Hence, too, the caution with which we should proceed to subject a patient to a course of mercury.

One word now with respect to the treatment of chancres. I think it is a matter of the utmost importance to the medical man, as well as to the patient, that chancres should be seen and treated in the very commencement, that is from two to four or six days after their appearance. Like the effects of many animal poisons, they are at first merely a local disease, and seldom affect the constitution, until they have been for some time in existence. In the beginning they produce local irritation, but if neglected, may give rise to constitutional affection. Hence the importance of being treated from the commencement, and to this circumstance I attribute the chief part of the success that atttended Dr. Roe's practice, and the rare occurrrence of secondary symptoms among the men entrusted to his care. I feel convinced that chancre, if seen shortly after its appearance, may, in eight cases out of ten, be treated safely and successfully without a single grain of mercury.

There are very few animal poisons which may not be arrested and destroyed at the point of inoculation, if treated properly. I feel fully convinced, that if you were to take a vaccine vesicle, and destroy it with nitrate of silver shortly after it has made its appearance, the virus would not affect the constitution, and that the child would not be protected from the danger of infection from small-pox. Burn the whole vesicle, it will heal up like any other part, and the child will not be safe from infection. You may smother the disease while it is merely local, and before the constitution is affected. Such at least appears to be the case with many animal poisons, and in particular with regard to the venereal.

As it is extremely desirable to arrest the local progress of chancre, many methods of accomplishing this object have been devised, among which none appear more certain or efficacious than the application of escharotics. If the disease be detected in its very early stage, before the matrix pimple has burst, or immediately after that event, the destruction of the local disease proves, in the great majority of cases, a perfect protection against constitutional sequelæ. When the chancrous ulceration has once commenced, and has been allowed to remain unchecked for one, or two, or three days, it is still most desirable to extirpate the local malady, and the result will generally be successful. The chance of protecting the constitution diminishes in proportion as the operation is deferred, but we want data to enable us to calculate at what period it ceases to be at all protective; that period, probably, varies in different cases.

Be this as it may, it is an essential point in practice to get rid of the primary sore as speedily as possible: how it is best to effect this object is a subject which requires a few remarks. The usual mode of treating small sores, whose diameter does not exceed that of a common stick of lunar caustic, is to apply the latter in substance, so as to produce a small eschar of the required size: this method seldom fails, but is attended with the disadvantage that it often gives rise to sympathetic bubo, as the caustic is not unfrequently used with too little caution. I had, accordingly, given up the use of the solid caustic, except where the pimple or ulcer is very small, requiring merely a slight touch of the pointed pencil. Many practitioners lean too heavily on the pencil during its application, and keep it too long applied, and consequently, the resulting inflammation and eschar are far more considerable than are necessary, and particularly more likely to produce bubo.

When the sore is so large that the diameter of its surface equals, or nearly equals a line, it is already too extensive for the application of the solid caustic without incurring the risk of bubo. Under these circumstances, or, *à fortiori*, when the sore is still larger, I use the following method:—provide yourself with a common-sized, nicely-pointed camel's-hair pencil, and a solution of lunar caustic, twenty grains to the ounce. Pour a drop or two of this on the cover of a book, or on the table, and dipping the brush in a basin of water, cleanse the surface of the sore with it. Dry the sore then completely with a piece of lint, and, rinsing the brush, squeeze out the chief part of the water, and, pointing the brush, you may then dip the extreme point of it in the drop of caustic solution, so as to take up the smallest possible quantity of fluid, which you may then apply to the centre of the sore. When it has done acting, we may readily judge, by the appearance of the surface, whether enough has been applied, for the whole surface must be whitened; but it is not, as is usually imagined, proper to burn out the edges. It may be necessary to dip the end of the brush in the solution, and apply it to the sore a second or even a third time, pausing to observe the effects of such application. By proceeding thus, we destroy the diseased surface, and do not produce any inflammation likely to give rise to bubo.

Some practitioners are much bolder, and use the solid caustic much more freely, desiring the patient to keep the part poulticed; but their mode of proceeding is very objectionable. When the solution has been properly and cautiously applied, no dressing to the part is required, except a bit of lint or charpie. In some cases, it is better to use as an escharotic the nitrate of copper, which may be employed in the form of concentrated solution, obtained by allowing the solid salt to deliquesce. Here the camel's-hair pencil and the same precautions are required.

After cauterizing the surface of a chancre, I have frequently applied a little of the fur or felt of hat to the ulcer, and directed the patient not to remove it, if it adhered to the surface, which it will sometimes do, forming a scab that does not drop off until the sore is quite healed. Although we may not have recourse to applications decidedly escharotic, (which is the surer way,) yet I think the early and diligent use of stimulating lotions of lead, sulphate of copper, and sulphate of zinc washes, serve, to a certain degree, to protect the constitution. The fact is, that chancres so treated in the very beginning, and thus altered, and caused to assume a healing process, cease to be so likely to infect the system either of the individual himself, or of females with whom he may have connection. A similar remark applies to gonorrhœa; an astringent injection, used several times immediately before connection, will, for the time, so alter the nature of the urethral secretion, that it will cease to be infectious, although it may become so in half an hour or an hour afterwards.—*Lon. Med. Gaz.*

---

*Preparations of Gold in Scrofula.*—The preparations of gold have been recently employed by M. Baudelocque, at the Hospital of Sick Children, and by M. Velpeau, at La Charité, in cases of scrofula. At the former of these two hospitals the preparations of gold have been administered in enormous doses. M. Baudelocque has given the hydrochlorate and stannate of gold, in doses of from ten to twelve grains, without producing any effect on the disease; and what is still more remarkable, without any apparent injury to the constitution of the children submitted to experiment. The oxide of gold, prepared by potash, was administered in as high as twenty grains during the day. At La Charité M. Velpeau has given fifteen, eighteen, and twenty grains of the hydrochlorate and oxide of gold during the day. Higher doses were not tried, merely on account of the great expense of the medicine. The above results are curious, because the preparations of gold have hitherto been regarded as extremely poisonous. M. Orfila says that the hydrochlorate of gold is infinitely more active than the corrosive sublimate, and M. Devergie mentions that at the dose of one-tenth to one-twentieth of a grain, it produces more or less inflammation of the gastro-intestinal mucous membrane.—*L'Exp.*

---

*Dessault's Treatment of Hospital Erysipelas.*—As soon as ever Dessault perceived any trace of the development of erysipelas after wounds, operations, &c., he immediately administered to the patient one grain of tartar emetic in a large quantity of water. The unfavourable symptoms immediately diminished after the administration of the draught, and sometimes disappeared altogether, even when the only effect of the medicine was to increase the urine, and augment the cutaneous transpiration; were the symptoms more obstinate, he then gave the draught two or three times, or oftener; as the fever disappeared, and nothing remained but some unpleasant bitter taste in the mouth, he completed the cure with one or two purgatives. Dessault assures us that he never met with a case of hospital erysipelas (and there were many of them at the Hôtel Dieu in his time) which resisted this method of treatment. He remarked that the disease was always most obstinate and severe in persons who had been bled several times.—*Med. Gaz.*

